# HIV-HPV interactions via extracellular vesicles among tobacco smokers and nonsmokers

**DOI:** 10.3389/ebm.2025.10687

**Published:** 2026-01-21

**Authors:** Namita Sinha, Laree Hiser, Sandip Godse, Lina Zhou, Zhanserik Shynykul, Carolann Risley, Theodore Cory, Santosh Kumar

**Affiliations:** 1 Department of Pharmaceutical Sciences, College of Pharmacy, The University of Tennessee, Health Science Center, Memphis, TN, United States; 2 Department of Cell and Molecular Biology, School of Nursing, Cancer Center and Research Institute, School of Medicine, The University of Mississippi Medical Center, Jackson, MS, United States; 3 Department of Clinical Pharmacy and Translational Sciences, College of Pharmacy, The University of Tennessee, Health Science Center, Memphis, TN, United States

**Keywords:** HIV, HPV, tobacco smokers, extracellular vesicles, inflammation

## Abstract

Human Immunodeficiency Virus (HIV) and Human Papillomavirus (HPV) co-infections are significantly prevalent, especially among African Americans (AA), a situation further compounded by the prevalence of tobacco smoking. Extracellular vesicles (EVs) are integral to the mechanisms of viral pathogenesis, as they are pivotal in the modulation of immune responses and the inflammatory process. This research study examines the varying concentrations of EVs, their associated biomarkers, and the cytokine/chemokine profiles present in plasma obtained from individuals infected with HIV and those coinfected with HIV and HPV, with particular emphasis on the ramifications of smoking behavior. Our findings revealed that HIV infection markedly elevates EV formation and modifies their protein composition, whereas HPV co-infection does not significantly augment EV levels but does influence the specific cytokine packaging. Notably, monocyte chemoattractant protein-1 (MCP-1 or CCL2) and Regulated upon Activation, Normal T cell Expressed and presumably Secreted (RANTES or CCL5) exhibited substantial enrichment in EVs derived from individuals coinfected with HIV and HPV, implying a potential role of EVs in immune modulation related to viral persistence. Importantly, smoking was found to affect EV characteristics, resulting in an increase in EV size and the packaging of inflammatory mediators, such as MCP-1 and interleukin-18 (IL-18), from plasma into EVs in HIV- and/or HIV+HPV-infected samples. This observation suggests that oxidative stress induced by smoking may intensify immune dysregulation through modifications in EV-mediated cytokine signaling pathways. Nevertheless, smoking did not exhibit a significant impact on the expression of EV marker proteins or the overall levels of EVs. These outcomes underscore the intricate interactions between HIV, HPV, and/or smoking in influencing the immune milieu via EVs. Further comprehensive understanding of the role of EVs in the context of these viral infections could yield valuable insights into potential biomarkers for disease progression and new therapeutic strategies.

## Graphical Abstract

**Figure:**
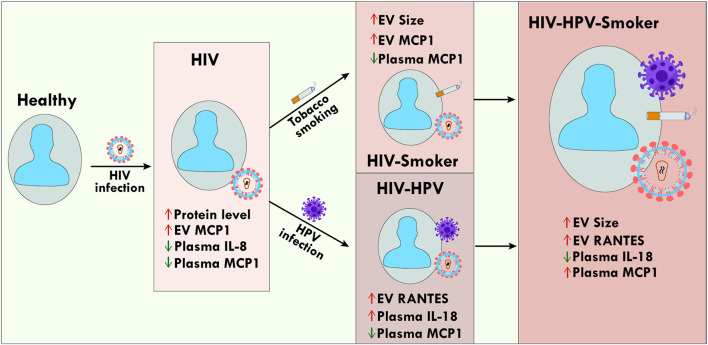

## Impact statement

Despite notable progress in the management of HIV through the implementation of antiretroviral therapies, the incidence rates and prevalence of HIV-associated neurocognitive disorders (HAND) continue to escalate, with a disproportionate impact on African Americans (AA). Furthermore, human papillomavirus (HPV), a highly endemic sexually transmitted infection, exacerbates the overall disease burden, particularly amongst individuals with compromised immune systems. Epidemiological data indicates a bidirectional interplay between HIV and HPV infections, potentially mediated by oxidative stress and inflammatory mechanisms. Tobacco consumption, which is notably higher among individuals living with HIV, further intensifies immune suppression, oxidative stress, and inflammatory responses, thereby facilitating disease progression. Extracellular vesicles (EVs) act as pivotal mediators of intercellular communication and may influence the pathogenesis of both HIV and HPV. This investigation examines potential HIV-HPV interactions via EVs by measuring the variations in the levels of EVs and their associated oxidative stress biomarkers and inflammatory cytokines and chemokines in individuals coinfected with HIV and HPV, with a particular focus on smokers.

## Introduction

Despite considerable progress in the management of Human Immunodeficiency Virus (HIV) facilitated by antiretroviral therapies, rates of infection persist in an upward trajectory both on a global scale and within the United States [1]. Notably, African Americans (AA) or Black people are disproportionately affected by HIV infections, and due to HIV-related conditions, they experience relatively high mortality. Recent data from the Centers for Disease Control and Prevention (CDC) indicate that although AA constitute 12% of the overall population in the United States, they account for 44% of newly diagnosed HIV infections [2]. Furthermore, HIV-associated neurocognitive disorders (HAND), particularly mild and asymptomatic forms, affect an estimated 50% of individuals living with HIV [3, 4]. Given that over half of the HIV-positive population is aged 50 and older, the prevalence of HAND is expected to rise in the coming years [5].

Human papillomavirus (HPV) is the most common sexually transmitted pathogen that causes anogenital cancers, with approximately 80% of people having acquired an HPV infection by age 50. Similar to HIV infection, the prevalence of anogenital HPV is also higher in AA than Caucasian Americans [6]. When HPV infection persists, it can lead to the development of anogenital and other cancers [7, 8]. Among more than 100 types of HPV, the most prevalent high risk types of HPV are HPV16 and 18 [7–9], which have been detected in ∼70% of cervical cancers [8, 10].

Epidemiological data suggest that the risk of HIV infection is increased by ∼2-fold in patients who are infected by HPV, and the risk of HPV infection is also increased in HIV seropositive patients [11–14]. It is possible that crosstalk exists between HPV- and HIV-infected cells, since HPV infection strongly correlates with immunosuppression, which is the hallmark of HIV infection. Therefore, investigation of biological interactions between HPV- and HIV-infected cells is important towards finding effective interventions for patients with this comorbidity. Previously, we have shown that supernatants from HPV-infected cells increase HIV replication via cytochrome P450 (CYP) and oxidative stress pathways when applied to HIV-infected cells *in vitro* [15].

Tobacco smoking is more prevalent in people living with HIV compared to the general population [16]. It is well established that cigarette smoke and tobacco constituents differentially regulate immune activation, inflammation, and oxidative stress [7, 8], leading to HIV pathogenesis [9–14]. Our own studies [17–19] have confirmed this. Persistent HIV pathogenesis in tobacco users exacerbates HAND [15–17]. Similarly, tobacco smoking, including second-hand smoking, is associated with a high risk of HPV infection and HPV-related pathogenesis, including cervical cancer [20, 21]. Further this study has shown that the combination of HPV and cigarette smoke promotes superoxide dismutase 2 (SOD2) alterations, leading to increased DNA damage and HPV pathogenesis. An epidemiological study also shows an association between CYP1A1, which activates tobacco constituents, and an increased susceptibility for HPV-induced precancer in the uterine cervix [22]. Recently, we have shown the role of inflammatory pathways via tumor necrosis factor α (TNF-α), nuclear factor kappa-light-chain-enhancer of activated B cells (NF-κB), and interleukin-6 (IL-6) in smoking-induced HPV pathogenesis, including HPV16 E6/E7 oncogenes and epithelial-mesenchymal transition markers in cervical cancer cells [23].

Literature reports and our studies [24] suggest that redox balance and oxidative stress are promoting factors in HPV-initiated pathogenesis [25, 26]. HPV infection can modulate the host cell redox homeostasis to favor infection and possibly alter cellular metabolism to enhance HIV pathogenesis [24–28]. Furthermore, increased inflammatory markers found in women with persistent HPV infection could lead to decreased immune functions and increased inflammation [29, 30], which are hallmarks of HIV pathogenesis.

Extracellular vesicles (EVs) play a significant role in the progression and modulation of both HIV and HPV infections, resulting in cellular pathogenesis including cervical cancers, by facilitating intercellular communication between infected and uninfected cells [18, 19, 31]. Upon HIV infection, EVs derived from infected cells can carry viral proteins, RNA, and oxidative and inflammatory mediators enhancing viral spread, immune activation, and chronic inflammation [20] (PMID: 38655385). Recent studies have shown that EVs in HIV-infected cells can package specific biomolecules including miRNA and oxidative and inflammatory agents upon exposure to drugs of abuse including tobacco smoking [21, 22]. Similarly, upon HPV infection, EVs can transport viral oncogenes such as E6 and E7, contributing to immune evasion and cancer progression, particularly in cervical and head and neck cancers [23–25].

It is conceivable that EVs that are differentially packed with components of the oxidative stress pathway, such as antioxidant enzymes (AOEs) and inflammatory cytokines and chemokines derived from HPV-infected cells, are circulated in plasma so that the contents are delivered to HIV-infected cells. The enhanced levels of oxidative stress and inflammation may thus contribute to enhanced HIV pathogenesis in HPV+HIV coinfected cells. EVs are small nanovesicles that can package and transport diverse biological cargo, such as proteins, mRNA, and miRNA, to distant cells, thus serving both as diagnostic biomarkers [32, 33] and as potential therapeutic targets [34]. We have shown elevated levels of HPV cell-derived EVs containing CYP1A1, CYP2A6, SOD1, and HPV oncoproteins (HPV16 E6) [24].

In this study, our objective is to examine differential levels of EVs and EV biomarkers, particularly oxidative stress and inflammatory cytokines/chemokines, in plasma derived from healthy, HIV-positive, and HIV+HPV-positive subjects. Our hypothesis is that the levels of EVs and EV components will differ in plasma derived from HIV+HPV-positive subjects compared to subjects infected with only HIV or neither virus. We further hypothesize that these EV components will differ in HIV+HPV-positive subjects who are tobacco smokers compared to nonsmokers. Since HIV and HPV are more prevalent in the AA population, and HPV is more prevalent in women, we examined the levels of plasma EV and their components in AA women, comparing smokers to nonsmokers.

## Materials and methods

### Sample and study design

We conducted a case-control study by recruiting participants from five distinct groups: healthy nonsmokers, HIV-positive nonsmokers, HIV and HPV coinfected nonsmokers, HIV-positive smokers, and HIV and HPV coinfected smokers (Table 1). Written informed consent was obtained from all participants, and institutional review board approval was secured at each site—The University of Tennessee Health Science Center and The University of Mississippi Medical Center. The study sample consisted of 5-6 AA women in each group ranging between 29 and 68 years of age (Table 1). Apparently, neither HPV coinfection nor smoking appeared to significantly impact CD4 counts, likely due to the controlled viral load since they were on ART (Table 1). The HIV viral load was ≤21 copies/mL with two exceptions: one participant in the HIV^+^/HPV^−^ group had a viral load of 36 copies/mL and another one in the HIV^+^/HPV^+^ group had a viral load of 586 copies/mL. Since they were on ART, the HIV viral load was undetectable in most women: 4 of 6 in the HIV^+^/HPV^−^ group who had never smoked, 3 of 4 HIV^+^/HPV^−^ ever smokers, 3 of 5 HIV^+^/HPV^+^ nonsmokers, and 2 of 5 in the HIV^+^/HPV^+^ ever smoker group.

**TABLE 1:** Sample characteristics.

Description	Age	CD4 counts (cells/mm^3^)	Smoking status	HIV status	HPV status
Healthy nonsmoker (n = 6)	24–65	​	Never smoked cigarettes	Negative	Negative
HIV^+^/HPV^−^ nonsmoker (n = 6)	30–55	739 ± 269	Never smoked cigarettes	Positive	Negative
HIV^+^/HPV^+^ nonsmoker (n = 5)	35–64	694 ± 314	Never smoked cigarettes	Positive	Positive
HIV^+^/HPV^−^ smoker (n = 6)	45–68	754 ± 254	Currently smoke cigarettes some days of the week	Positive	Negative
HIV^+^/HPV^+^ smoker (n = 5)	29–65	813 ± 213	Currently smoke cigarettes some days of the week	Positive	Positive

For EV characterization and Western blot analysis, we used samples from four randomly selected subjects per group, and for cytokine and chemokine analysis, samples from five to six subjects per group were analyzed. In this study, we did not recruit a group with HPV infection alone or a healthy smoker group because our goal was to determine how HPV infection, with or without tobacco smoking, alters EV constituents specifically within the context of HIV infection.

A chart review was conducted to determine inclusion and exclusion criteria for participants’ recruitment. The inclusion criteria were: (A) Healthy group—generally healthy individuals, as determined by a standard history and physical exam, with no evidence of HPV on an anal and/or cervical Papanicolaou (Pap) test within the last 3 months and a negative HIV test within the last 6 months; (B) HIV^+^/HPV^−^—HIV-positive individuals with a manageable CD4 count (>400 cells/μL) based on the CDC’s 2008 Stage II HIV-1 classification, drawn within the past 3 months, and negative for high-risk HPV on an anal or cervical Pap test within the past 3 months; (C) HIV^+^/HPV^+^—HIV-positive individuals with a manageable CD4 count tested within the past 3 months and positive for a high-risk HPV type based on an anal or cervical Pap smear within the past 3 months; and (D) Smoker groups—individuals who currently smoke cigarettes every day of the week or on some days of the week. The exclusion criteria included children, pregnant or lactating women, current or former smokers in the nonsmoker group, and individuals diagnosed with tuberculosis or hepatitis A, B, or C.

### Isolation and characterization of plasma EVs

Buffy coats or blood samples from freshly drawn blood were processed to isolate plasma. Approximately 50 mL of blood was collected in 3 vacutainer tubes with potassium EDTA using a 21-gauge needle. Centrifugation at 750 × *g* for 10 min at 4 °C was performed within 15 min of blood collection, and plasma was collected and stored at −80 °C within 3 h of blood draw. EVs were freshly extracted from plasma (50 µL) specimens utilizing a modified methodology derived from previously established protocols [26, 34, 35], employing a commercially available precipitation reagent (Invitrogen Catalog number 4484450). The method yields EVs with minimal plasma, including HIV particles, or cellular contaminations, which are not likely to interfere with our analysis. Initially, plasma underwent filtration through a 0.22 μm filter followed by centrifugation at 10,000 x *g* for a duration of 20 min to eliminate larger particulates. The resultant supernatant was combined with 0.5 volumes of phosphate-buffered saline (PBS) and 0.2 volumes of the exosome precipitation reagent, then incubated at ambient temperature for 10 min, followed by centrifugation at 10,000 x *g* for 5 min to obtain the EV pellet. The EV pellet was resuspended in 100 µL RIPA buffer or PBS as needed for the subsequent experiments. We measured the size and zeta potential of EVs by dynamic light scattering using a Zetasizer Nano-ZS (Malvern Instruments Inc, Malvern, UK).

### Western blot analysis

Isolated EVs were lysed with RIPA buffer including protease inhibitors, and protein concentration was determined using the Pierce BCA Protein Assay Kit (ThermoFisher Scientific, Cat. No. 23225). Equal protein amounts (10 µg) were loaded on polyacrylamide gels, electrophoresed, and transferred onto PVDF membranes. Membranes were blocked in LI-COR blocking buffer for one hour and incubated overnight at 4 °C with primary antibodies specific to CD63 (1:500, Cat. No. 25682-1-AP, Proteintech), CD9 (1:500, Cat. No. 60232-1-1g, Proteintech), Alix (1:500, Cat. No. sc-53538, Santa Cruz Biotechnology), IL-6 (1:400, Cat. No. 21865-1-AP, Proteintech), IL-18 (1:400, Cat. No. 10663-1-AP, Proteintech), MCP-1 (1:400, Cat. No. 66272-1-1g, Proteintech), SOD1 (1:1000, Cat No. sc-101523, Santa Cruz Biotechnology), or catalase (1:500, Cat. No. 21260-1-AP, Proteintech). After washing, membranes were incubated with IRDye-conjugated secondary antibodies (1:10,000, Cat. No. 926-32211 for anti-mouse and 926-68072 for anti-rabbit, LI-COR Biosciences) for one hour at room temperature in the dark. Protein bands were visualized using the LI-COR Scanner and quantified using LI-COR Image Studio Software (v5.2, LI-COR Biosciences).

### Multiplex ELISA

Cytokine and chemokine concentrations, encompassing both pro-inflammatory and anti-inflammatory mediators, were evaluated in plasma-derived EVs and plasma utilizing a tailored human 9-Plex ProcartaPlex™ multiplex immunoassay (ThermoFisher Scientific) [27, 28]. The method yields minimal EV cytokines/chemokines contamination from plasma samples, which is not likely to interfere with our analysis. A 50 µL plasma sample and equivalent amount of EVs isolated from 50 µL plasma were used for ELISA. Samples and standards were incubated in a magnetic 96-well enzyme-linked immunoassay (ELISA) plate for one hour at ambient temperature, followed by a series of washing steps. The quantification of cytokine and chemokine concentrations was carried out according to the assay protocols, and the resulting concentrations were reported in pg/mL. EV packaging efficiency was calculated by dividing the cytokine/chemokine value in EVs by the corresponding plasma (free cytokines/chemokines) plus EV cytokine/chemokine values and multiplying by 100 (EV packaging = EV packaging (%) = [EV / (plasma + EV)] × 100. Plasma samples used for ELISA (50 μL, without detergent treatment) contained both free and EV-associated cytokines/chemokines, but only free cytokines/chemokines were quantified. For simplicity, we write this as plasma cytokines/chemokines in results and discussion. In contrast, EV pellets were resuspended in a detergent-containing buffer, allowing release of EV-encapsulated cytokines/chemokines. Some degree of cross-contamination is possible. For example, plasma cytokines/chemokines may contain EV surface cytokines/chemokines, or EV cytokines/chemokines may carry cytokines/chemokines from plasma fraction. Although the absolute percentage of cytokines/chemokines packaged within EVs may therefore be imprecise, the relative comparison of EV packaging among different groups remains valid.

### Statistical analysis

All outcomes are shown as the mean ± standard error of the mean (SEM) from n = ≥4 subjects per group. Comparisons of statistical data between experimental groups were conducted using one-way ANOVA with Tukey’s post-hoc test applied for multiple comparisons. A *p*-value of less than 0.05 was considered statistically significant, with significance levels denoted as follows: *p < 0.05, **p < 0.01, ***p < 0.001, ****p < 0.0001.

## Results

### Characterization of plasma EVs from healthy nonsmokers and HIV and HIV+HPV individuals in nonsmoker and smoker groups

**FIGURE 1:**
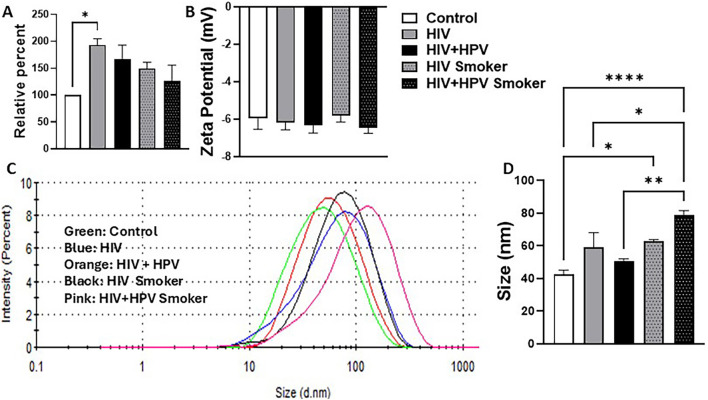
Characterization of plasma EVs from healthy nonsmokers and HIV and HIV+HPV individuals in nonsmoker and smoker groups. EVs were isolated using filtration and precipitation as described in Materials and Methods. EVs were characterized using. **(A)** Quantification of total EV proteins presented as relative percent, **(B)** Zeta potential, **(C)** Size distribution (intensity-weighted), and **(D)** Mean size. The EV proteins were quantified using Pierce BCA Protein Assay Kit, and the size and zeta potential of EVs were measured and analyzed using the dynamic light scattering (DLS) method. Statistical analysis was performed by one-way ANOVA with Tukey’s *post hoc* test. Values are shown as mean ± SEM (*n* = 4/group). *p < 0.05, **p < 0.01, ****p < 0.0001.

Our results demonstrated that the total plasma-derived EV protein levels were significantly elevated in the HIV-positive, nonsmoker group (Figure 1A, *p < 0.05) compared to the healthy control. However, the presence of HPV co-infection in the HIV-positive nonsmoker group did not result in a further increase in EV protein levels. Similarly, in both the HIV-positive and the HIV+HPV-positive smoker groups, tobacco smoking did not lead to a further increase in EV protein levels. Although these groups showed a pattern of increase in EV protein levels compared to healthy groups, the increase was not statistically significant. Thus, HIV infection is the primary driver of elevated EV protein levels, whereas HPV co-infection or smoking does not contribute to further increases. Smoking markedly decreased the plasma EV protein levels in both HIV-positive and HIV+HPV-positive individuals, although the results were not statistically significant.

The zeta potential of plasma EVs remained unchanged across all experimental groups, including HIV-positive and HIV+HPV-positive nonsmoker and smoker groups, indicating a consistent surface charge distribution relative to the healthy control (Figure 1B). This suggests that neither HIV nor HPV co-infection, alone or in combination with smoking, significantly altered the electrostatic properties of EVs.

The plasma EV size exhibited notable differences across groups (Figures 1C,D). Compared to the healthy control (42.65 ± 2.60 nm), the HIV-positive (59.13 ± 9.012 nm) and HIV+HPV-positive (50.57 ± 1.61 nm) nonsmoker groups showed a modest increase in EV size, but they were not statistically significant. More pronounced differences were observed in the smoker subgroups, where the HIV-positive smoker group (62.77 ± 1.17 nm) had significantly larger EVs than the control, and the HIV+HPV-positive smoker group (78.97 ± 2.77 nm) displayed the most substantial increase (Figures 1C,D). Statistical comparisons indicate that the HIV+HPV-positive smoker group exhibited a significantly larger EV size than both the HIV-positive and HIV+HPV nonsmoker groups. The finding that smoking in the context of HIV and HPV co-infection contributes to a significant increase in EV size suggests potential alterations in EV biogenesis or cargo composition (Figures 1C,D). There is mixed correlation between EV protein levels (Figure 1A) and EV size (Figures 1C,D) for most groups suggest that the increase in EV protein levels could be a result of either increased EV size or increased EV numbers. Compared to healthy individuals, HIV-positive non-smokers exhibited an increase in EV protein levels without a significant change in EV size. In contrast, among HIV-HPV coinfected individuals, smokers showed an increase in EV size compared to nonsmokers, without a corresponding change in EV protein levels.

### Relative levels of plasma-derived EV negative and positive marker proteins in healthy nonsmokers and HIV and HIV+HPV individuals in nonsmoker and smoker groups

**FIGURE 2:**
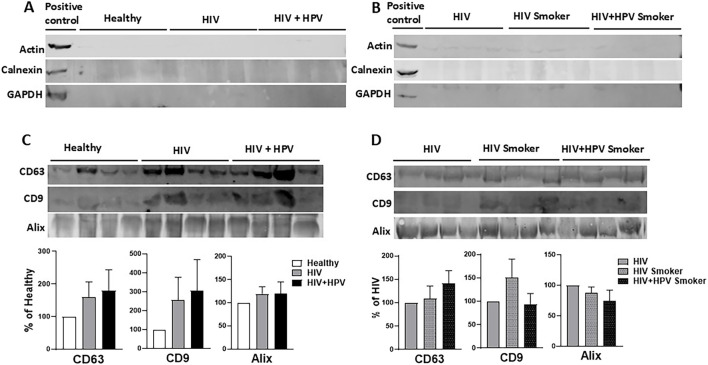
Relative levels of plasma-derived EV negative and positive marker proteins. **(A)** Analysis of EV negative marker proteins actin, calnexin, and GAPDH from plasma-derived EVs in healthy, HIV, and HIV+HPV nonsmoker groups. **(B)** Analysis of EV negative marker proteins actin, calnexin, and GAPDH from plasma-derived EVs in HIV nonsmoker and HIV and HIV+HPV smoker groups. **(C)** Analysis of EV marker proteins CD63, CD9, and Alix from plasma-derived EVs in healthy, HIV, and HIV+HPV nonsmoker groups. **(D)** Analysis of EV marker proteins CD63, CD9, and Alix from plasma-derived EVs in HIV nonsmoker and HIV and HIV+HPV smoker groups. Western blot of EV marker proteins and their quantitative analyses were performed as described in Materials and Methods. Values are expressed as mean ± SEM (*n* = 4/group).

**FIGURE 3:**
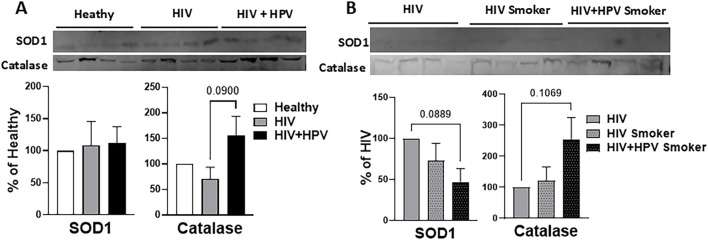
Relative levels of plasma-derived EV antioxidant enzymes. **(A)** Analysis of SOD1 and catalase from plasma-derived EVs in healthy, HIV, and HIV+HPV nonsmoker groups. **(B)** Analysis of SOD1 and catalase from plasma-derived EVs in HIV nonsmoker and HIV and HIV+HPV smoker groups. Western blot of EV SOD1 and catalase and their quantitative analysis were performed as described in Materials and Methods. Statistical analysis was performed by one-way ANOVA with Tukey’s *post hoc* test. Values are expressed as mean ± SEM (n = 4/group). Because no comparisons reached statistical significance at p < 0.05, actual p-values ≤0.1 are reported.

**FIGURE 4:**
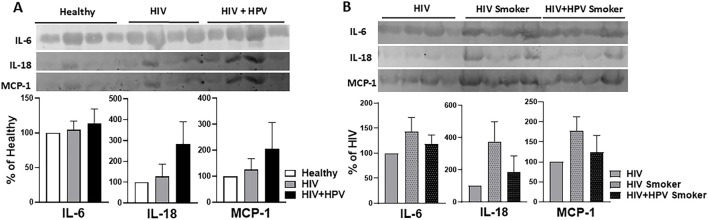
Relative levels of plasma-derived EV cytokines and chemokines. **(A)** Analysis of cytokines IL-6 and IL-18 and chemokine MCP-1 from plasma-derived EVs in healthy, HIV, and HIV+HPV in nonsmoker groups. **(B)** Analysis of cytokines IL-6 and IL-18 and chemokine MCP-1 from plasma-derived EVs in HIV nonsmoker and HIV and HIV+HPV smoker groups. Western blot of EV cytokines and chemokines and their quantitative analysis were performed as described in Materials and Methods. Statistical analysis was performed by one-way ANOVA with Tukey’s *post hoc* test. Values are expressed as mean ± SEM (*n* = 4/group). No comparisons reached statistical significance at p < 0.05.

EV negative marker proteins (actin, calnexin, and GAPDH) and EV marker proteins (CD63, CD9, and Alix) were examined in plasma samples from the five different groups. Western blot analysis was performed to verify the existence or non-existence of these markers in the EV fractions, affirming the legitimacy of the isolated vesicles (Figure 2). As expected, while actin, calnexin, and GAPDH were present in positive control (cellular fractions), these proteins were absent or negligible in EV fractions of all the groups (Figures 1A,B). In comparison to the healthy nonsmokers, HIV infection resulted in a marked increase in CD63 and CD9, but not Alix levels in EVs in the HIV-positive and HIV+HPV-positive nonsmoker groups, suggesting that HIV infection could alter EV composition (Figure 2C). However, the changes did not reach statistical significance. On the other hand, compared to HIV-positive nonsmoker groups, the levels of none of the EV marker proteins were substantially altered in HIV and HIV+HPV-positive smoker groups (Figure 2D). These findings indicate that although HIV and HPV infections may affect EV cargo by enhancing CD63 and CD9, smoking does not additionally modify these changes. It should be noted that because two separate Western blots were performed for the same HIV non-smoker samples in Figure 2A vs. Figure 2B and Figure 2C vs. Figure 2D, the observed differences in protein band intensities are attributable to inter-blot variability. Similarly, since these are two different blots, we cannot compare results between Figure 2C,D. These situations apply to the subsequent analysis in Figures 3, 4.

### Relative levels of plasma-derived EV antioxidant enzymes in healthy nonsmokers and HIV and HIV+HPV individuals in nonsmoker and smoker groups

Next, we examined the levels of the common plasma EV oxidative stress marker proteins, SOD1 and catalase, using Western blot analysis (Figures 3A,B). These antioxidant enzymes (AOEs) can detoxify oxidants and reduce oxidative stress. Their presence in plasma EVs suggests that AOEs originating from one tissue or organ could lead to altered oxidative stress in distant recipient tissues or organs via systemic circulation. Although not statistically significant, the levels of EV catalase appear to increase in HIV+HPV nonsmokers in comparison to healthy controls (Figure 3A). Our results also showed that compared to healthy nonsmokers with HIV, SOD1 and catalase had opposite trends; SOD1 decreased, and catalase increased in both the HIV and HIV+HPV smoker groups, although the results were not statistically significant (Figure 3B). These findings suggest that while HIV+HPV coinfections impact catalase levels in nonsmokers, both HIV and HIV+HPV infections impact both SOD1 and catalase in smokers.

### Relative levels of plasma-derived EV cytokines and chemokines in healthy nonsmokers and HIV and HIV+HPV individuals in nonsmoker and smoker groups

Similarly, important proinflammatory interleukins (IL-6 and IL-18) and a chemokine [monocyte chemoattractant protein-1 MCP-1 (also known as CCL2)] linked to EVs, were examined in plasma EV samples using Western blot analysis (Figure 4). Their involvement in inflammatory signaling and presence in EVs could suggest systemic immune activation and modified intercellular communication in relation to HIV and HPV infections, especially upon tobacco smoking.

Compared to healthy nonsmokers, HIV+HPV coinfection resulted in a marked increase in the levels of IL-18 and MCP-1, but not IL-6, in plasma EVs in the HIV+HPV-positive nonsmoker group (Figure 4A). However, the changes did not reach statistical significance. Similarly, although not statistically significant, the levels of these EV cytokines and chemokines were markedly increased in HIV-positive and slightly increased in HIV+HPV-positive smoker groups compared to the HIV nonsmoker group (Figure 4B). These findings suggest that while HIV+HPV coinfection may only increase EV cytokine and chemokine levels in nonsmoking groups, HIV infection may increase these levels in smoking groups.

### Relative cytokine and chemokine distributions between plasma and plasma-derived EVs in healthy and HIV and HIV+HPV individuals

**FIGURE 5:**
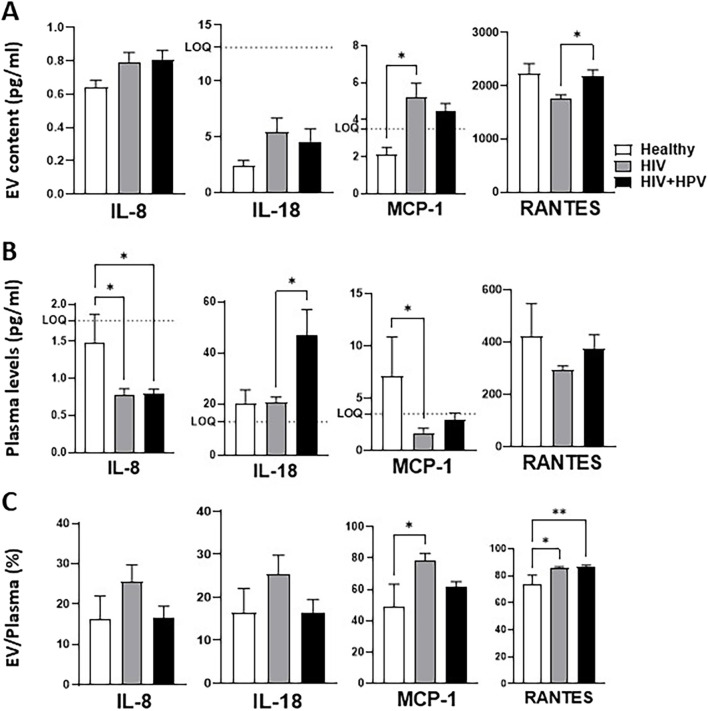
Relative cytokines and chemokines distributions between plasma and plasma-derived EVs in healthy, HIV, and HIV+HPV individuals. **(A)** Analysis of cytokines and chemokines in plasma-derived EVs of Healthy, HIV, and HIV+HPV. **(B)** Analysis of cytokines and chemokines in plasma of Healthy, HIV, and HIV+HPV. **(C)** Ratio of EV and plasma cytokines and chemokines in healthy, HIV, and HIV+HPV. Cytokine and chemokine levels were measured using a human 9-Plex ProcartaPlex™ multiplex immunoassay as described in Materials and Methods. The EV packaging efficiency was calculated by dividing the EV cytokine or chemokine values with that of plasma plus EV cytokine or chemokine values, multiplied by EV packaging (%) = [EV / (plasma + EV)] × 100. The ELISA measures only soluble or free plasma cytokines and chemokines. Statistical analysis was performed by one-way ANOVA with Tukey’s *post hoc* test. Values are expressed as mean ± SEM (*n* = 5–6/group). *p < 0.05.

Furthermore, we used the ELISA method to carry out the measurement and analysis of several plasma and EV-associated cytokines and chemokines, which are considered important in the context of HIV and HPV infections. In this analysis, we combined nonsmoker and smoker groups to assess the impact of HIV and HIV+HPV infections on EV and plasma cytokines/chemokines, regardless of smoking status. The analysis may offer insights into the packaging of plasma EV cytokines and chemokines and their possible function in immune activation and inflammation via intercellular communication upon HIV and HIV+HPV coinfections (Figure 5).

The IL-6 could not be detected by ELISA in this study. In comparison to the healthy nonsmokers, EV IL-8 exhibited an upward trend in both HIV and HIV+HPV infections, while plasma IL-8 was significantly decreased (Figures 5A,B). This may suggest a transfer of IL-8 from plasma to EVs, with a total packaging efficiency of 15–30% upon HIV and HIV+HPV coinfections, respectively. IL-18 displayed a rising trend in EV packaging in both HIV and HIV+HPV groups relative to healthy controls (Figure 5C). Interestingly, a significant increase in plasma IL-18 was observed in HIV+HPV coinfected compared to HIV. It is possible that overall IL-18 level is upregulated upon HIV and/or HIV+HPV infections, thereby increasing its levels in both EV and plasma. The total EV packaging efficiency of IL-18 was assessed at 15–25%, without a significant change in EV IL-18 packaging in any of the groups.

For the chemokine MCP-1, compared to healthy controls, a significant increase in EV protein was noted in the HIV group, and no further change was detected in the HIV+HPV group (Figure 5A). Conversely, compared to healthy controls, plasma MCP-1 concentrations were significantly and markedly decreased in individuals infected with HIV and HIV+HPV groups, respectively (Figure 5B). The total packaging efficiency of MCP-1 was 50% in healthy controls and was significantly and markedly increased in HIV (75%) and HIV+HPV (60%) groups, respectively (Figure 5C).

A significant rise in another chemokine RANTES, also known as CCL5, was seen in EVs of individuals coinfected with HIV and HPV when compared to the HIV-only group, whereas plasma RANTES did not vary significantly across the groups (Figures 5A,B). Perhaps HPV rescues the effect of HIV infection with regards to RANTES. Importantly, the total packaging efficiency of RANTES, a chemotactic factor for inflammatory and immune cells, in healthy controls was 75%, and was significantly increased in both HIV and HIV+HPV groups (85%) (Figure 5C).

Overall, these results show that proinflammatory cytokines IL-8 and IL-18 and chemokines RANTES and MCP-1 show a general trend of increased packaging in EVs and decreased levels in plasma in HIV-positive and/or HIV+HPV. Importantly, the high packaging percentage for RANTES and MCP-1 along with their increased packaging in HIV and/or HIV+HPV groups suggests a potential role for these chemoattractants in EV-mediated cell-cell interactions in HIV and/or HPV infected populations.

### Relative cytokine and chemokine distributions between plasma and plasma-derived EVs in HIV and HIV+HPV individuals in nonsmoker and smoker groups

**FIGURE 6:**
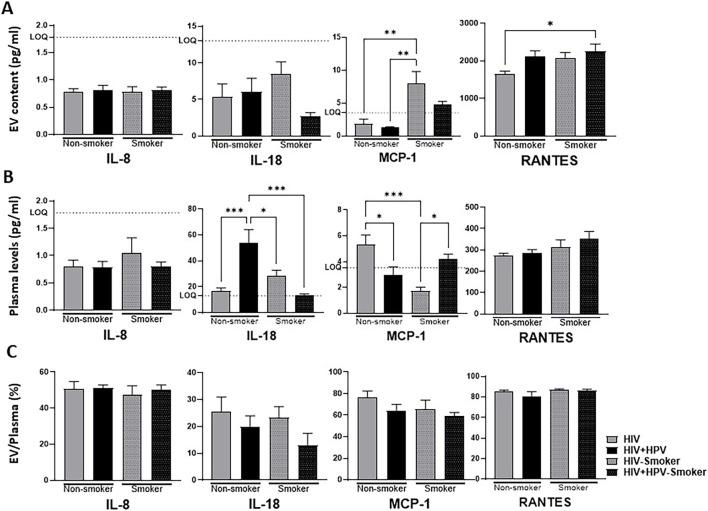
Relative cytokine and chemokine distributions between plasma and plasma-derived EVs in HIV and HIV+HPV individuals in nonsmoker and smoker groups. **(A)** Analysis of cytokines and chemokines in plasma-derived EVs of HIV and HIV+HPV individuals in nonsmoker and smoker groups. **(B)** Analysis of cytokines and chemokines in plasma of HIV and HIV+HPV individuals in nonsmoker and smoker groups. **(C)** Ratio of EV and plasma cytokines and chemokines in HIV and HIV+HPV individuals in nonsmoker and smoker groups. Cytokine and chemokine levels were measured using human 9-Plex ProcartaPlex™ multiplex immunoassay as described in Materials and Methods. The EV packaging efficiency was calculated by dividing the EV cytokines/chemokines values with that of plasma plus EV cytokines/chemokines values, multiplied by 100 (EV/(plasma+EV)x100). The ELISA measure soluble or free plasma cytokines/chemokines. Statistical analysis was performed by one-way ANOVA with Tukey’s *post hoc* test. Values are expressed as mean ± SEM (*n* = 5–6/group). *p < 0.05, **p < 0.01, ***p < 0.001.

The analysis of plasma cytokines and chemokines and EV-associated proinflammatory cytokines and chemokines was carried out in HIV and HIV+HPV groups, and they were compared between nonsmoker and smoker groups (Figure 6). We also analyzed EV packaging percentage of cytokines and chemokines in HIV and HIV+HPV individuals between nonsmoker and smoker groups.

The IL-6 could not be detected by ELISA in this study. One of the challenges of the IL-8 measurement was that the cytokine levels were below the lower level of quantification (LLQ), suggesting low detection sensitivity. Nevertheless, the analysis suggests that the levels of EV IL-8, plasma IL-8, and its packaging percentage (EV/plasma) did not significantly change in smokers compared to nonsmokers, irrespective of their infection status (HIV and/or HIV+HPV) (Figure 6). The analysis also showed a relatively high packaging (about 50%) across the groups (Figure 6C).

Similarly, compared to HIV infection, there was no significant change in EV IL-18 packaging by either HIV+HPV coinfection or smoking (Figure 6C). Interestingly, compared to HIV alone, HIV+HPV coinfections significantly increased plasma IL-18 in nonsmokers and markedly decreased the IL-18 plasma levels in smokers (Figure 6B). The overall packaging of IL-18 is relatively low (15–25%), compared to IL-8 packaging (about 50%), without a significant change between the groups (Figure 6C).

There was a significant and marked increase in EV MCP-1 packaging by HIV and HIV+HPV, respectively, in smokers compared to nonsmokers (Figure 6A). The increased EV packaging of MCP-1 in smoker groups, especially for HIV individuals, appears to be correlated with a significant decrease in the levels of MCP-1 in plasma. Interestingly, compared to HIV alone, HIV+HPV coinfections significantly decrease plasma MCP-1 in nonsmokers, but significantly increase plasma MCP-1 in smokers (Figure 6B). Similar to IL-18 and RANTES, the overall packaging of MCP-1 is also high (60–75%) across the groups, without a significant change between the groups (Figure 6C).

Similarly, the analysis showed a trend in increase in EV RANTES packaging by HIV+HPV coinfections, especially in smokers compared to nonsmokers and/or HIV alone. However, the plasma levels across the group were unaltered (Figures 6A,B). As expected, the EV packaging percentage was very high (>80%) in all the groups with no further increase in smokers for either HIV or HIV+HPV groups (Figure 6C).

Overall, the differential packaging of these cytokines and chemokines in EVs may indicate their role in modulating inflammation and immune responses under varying conditions. For example, MCP-1 shows increased packaging into EVs in smokers, and IL-18 appears to have the opposite trend, at least for HIV+HPV coinfections in nonsmokers vs. smokers (Figure 6A).

## Discussion

Tobacco smoking is a recognized risk factor that aggravates the progression of both HIV and HPV, with a notably higher prevalence of smoking found in individuals with HIV than in the general population [29], [36]. Previous research, including our findings, has shown that cigarette smoke and components of tobacco lead to differential immune regulation, inflammation, and oxidative stress; particularly, increased inflammation and oxidative stress speed up HIV progression and worsen HAND [35], [9–14], [27, 28], [30–32]. Likewise, tobacco consumption has been closely associated with heightened vulnerability to HPV infection and more severe HPV-related diseases, such as cervical cancer [23]. Cigarette smoke has been demonstrated to cause changes in SOD2, which results in heightened DNA damage and fosters HPV-related cancer development [37]. Moreover, the activation of cytochrome P450 enzymes, especially CYP1A1, boosts the metabolic activation of carcinogens from tobacco, heightening the vulnerability to premalignant alterations in HPV-infected cells [38]. Considering that both HIV and HPV pathogenesis involve immune dysfunction and oxidative stress, it is reasonable to suggest that tobacco exposure exacerbates these pathogenic processes via EVs, which act as carriers for inflammatory mediators and molecules associated with oxidative stress.

The findings of our research suggest that HIV infection is a significant factor in raising EV protein levels, while coinfection with HPV and smoking do not lead to additional increases. These results are consistent with earlier studies, which have shown that HIV infection results in heightened EV secretion, probably because the virus takes control of the exosomal biogenesis pathway to promote viral spread and immune modulation [39, 40]. The absence of increased EV protein levels in individuals coinfected with HPV indicates that HPV does not independently influence EV secretion in relation to HIV. This aligns with earlier research indicating that HPV mainly affects the composition of EV cargo rather than the total production of EVs [41]. Additionally, the consistent zeta potential observed in all experimental groups indicates that the surface charge of EVs does not vary with infection status, aligning with previous studies demonstrating that EVs preserve their electrostatic characteristics for effective cellular uptake [42]. Nonetheless, EV size showed distinct variations, especially in smokers, with HIV and HPV coinfected smokers experiencing the largest rise. This indicates that smoking could affect EV biogenesis or change the molecular makeup of EVs. Earlier research has indicated that components of tobacco, such as nicotine and polycyclic aromatic hydrocarbons, increase EV secretion and modify their cargo by inducing oxidative stress and inflammation [43]. The significant rise in EV size among smokers coinfected with HIV and HPV may indicate a heightened inflammatory response, possibly leading to an acceleration in disease progression.

Examination of EV marker proteins emphasizes how HIV alters EV composition, as shown by elevated CD63 and CD9 levels in those infected with HIV, consistent with current research [40]. The tetraspanins CD63 and CD9 are often upregulated in EVs from HIV-infected cells, likely contributing to viral spread and evasion of the immune response [44]. HPV coinfection did not increase levels of CD63 and CD9, indicating that HPV does not significantly influence EV tetraspanin-associated cargo. This discovery aligns with earlier research indicating HPV’s capability to modify EV composition, specifically regarding the inclusion of oncogenic proteins, rather than impacting EV marker proteins [40]. Furthermore, the level of Alix, an essential factor in EV biogenesis, was consistent in all groups, suggesting that neither HIV nor HPV interferes with the basic mechanism of EV formation. Smoking had no effect on EV marker expression, which contradicts certain studies suggesting that oxidative stress from smoking might change EV profiles [43]. The absence of alterations in EV marker levels among smokers indicates that although smoking might enhance EV size, it does not affect EV biogenesis or markers associated with tetraspanins. These results emphasize the intricate nature of EV-mediated interactions in HIV and HPV infections, underscoring the differing impact of viral infections and external influences like smoking on EV traits. Additional studies are required to clarify how smoking-related modifications in EV size could relate to functional changes in EV cargo and their effects on disease progression.

The effect of coinfection with HIV and HPV on EV-related inflammatory cytokines and chemokines offers important insights into systemic immune activation and cell-to-cell communication. Our results indicate that although HIV infection by itself or together with HPV does not substantially change IL-6, IL-18, or MCP-1 levels in EVs, there is a noticeable trend towards heightened IL-18 and MCP-1 levels in individuals infected with HIV, particularly in those also infected with HPV. This finding is consistent with earlier research indicating that coinfection with HIV and HPV leads to heightened inflammatory responses, mainly through pathways associated with IL-18 and MCP-1, which play roles in monocyte attraction and immune activation [43]. Nevertheless, the lack of notable IL-6 upregulation indicates that HIV and HPV might selectively influence inflammatory cytokines via EVs, likely emphasizing pathways linked to chronic inflammation and immune dysfunction over acute-phase responses [42]. Interestingly, smoking seemed to reduce this inflammatory response, as IL-18 and MCP-1 levels in the smoker groups did not follow the same patterns seen in nonsmokers. This discovery indicates that oxidative stress linked to tobacco might inhibit or change the packaging of cytokines within EVs, which could interfere with immune signaling and lead to immune dysregulation in smokers coinfected with HIV and HPV [44].

Additional examination of cytokine distribution in plasma and EVs indicated a notable change in inflammatory mediators like IL-8, RANTES, and MCP-1, showing greater incorporation into EVs among HIV and HIV+HPV coinfected individuals, whereas plasma concentrations of these cytokines were significantly diminished. This indicates that viral infections could promote the entrapment of inflammatory cytokines within EVs, potentially serving as a regulatory mechanism to manage excessive systemic inflammation. Consistent with our findings, earlier studies demonstrate that EV-mediated cytokine transport is vital for regulating immune responses in chronic infections [45]. The notable rise in EV-related RANTES in individuals coinfected with HIV and HPV, while maintaining stable plasma levels, underscores the possible function of EVs in specifically modulating chemokine signaling, potentially aiding in the recruitment of immune cells to sites of infection. Furthermore, the noted rise in MCP-1 packaging efficiency in HIV and HIV+HPV groups (50–80%) corroborates previous findings suggesting that MCP-1 plays a crucial role in monocyte trafficking during HIV infection [40]. The results indicate that the compartmentalization of cytokines into EVs may serve as a method through which HIV and HPV influence immune signaling to avoid detection by the immune system or to promote viral persistence. Overall, our research emphasizes the significance of EVs in influencing inflammatory responses during HIV and HPV coinfections and points out the necessity for additional studies on their effects on disease advancement and possible therapeutic interventions.

The distinct packaging of proinflammatory cytokines in EVs, along with their plasma concentrations in individuals coinfected with HIV and HPV, underscores an essential mechanism in immune modulation and the development of viral diseases. Our results indicate that IL-8, MCP-1, IL-18, and RANTES show heightened encapsulation in EVs, while their plasma concentrations are markedly lower in individuals coinfected with HIV and HPV. The noted transition of IL-8 from plasma to EVs, demonstrating a packaging efficiency of 15–30%, implies that EVs could serve as reservoirs for inflammatory cytokines, promoting intercellular communication and boosting local immune responses while curbing systemic inflammation. This aligns with earlier research suggesting that EV-mediated cytokine transport contributes to immune cell recruitment and activation in chronic infections, such as HIV [46]. Significantly, RANTES showed the highest packaging efficiency (75–80%), especially in individuals coinfected with HIV and HPV, aligning with findings that associate RANTES with immune cell movement and inflammation during HIV infection [47]. The significant rise in MCP-1 packaging in HIV and HIV+HPV coinfections reinforces its involvement in monocyte recruitment and ongoing immune activation, a key feature of HIV pathogenesis [48]. The change in cytokine distribution implies that EVs are crucial in controlling inflammation in individuals infected with HIV and HIV+HPV by capturing cytokines from the plasma, potentially altering their biological impact on immune balance and disease advancement.

The influence of smoking on EV-mediated cytokine behavior in HIV and HIV+HPV infections highlights its role in altering inflammatory responses. In contrast to IL-8, which stayed the same, both RANTES and MCP-1 displayed elevated levels in smokers, while MCP-1 demonstrated a shift from plasma into EVs without affecting its packaging efficiency. These results are consistent with earlier studies that emphasize smoking’s contribution to chronic inflammation and immune system dysfunction in individuals with HIV [49]. The noted decrease in plasma IL-18 among smokers, coupled with no significant rise in EV-associated IL-18, indicates that smoking might either inhibit IL-18 production or promote its removal, thus altering inflammatory pathways in a manner distinct from that of nonsmokers. Previous research has demonstrated that cigarette smoke triggers oxidative stress and modifies cytokine release, which may clarify these inconsistencies [50]. Moreover, the rise in RANTES levels found in smokers, in both plasma and EVs, indicates that smoking boosts chemokine signaling, possibly worsening immune cell recruitment and leading to ongoing inflammation. Since RANTES is vital in HIV pathogenesis by facilitating viral replication and immune activation, these results emphasize the necessity for additional research on smoking-related alterations in EV-mediated inflammatory signaling regarding HIV and HPV coinfections. Our research collectively offers new perspectives on the roles of EV-mediated intercellular interactions leading to altered inflammation upon HIV and HIV+HPV infections, especially in tobacco smokers.

In summary, smoking seemed to change EV properties, enlarging EV size and changing cytokine distribution, particularly leading to a significant transfer of MCP-1 and IL-18 from plasma into EVs. The results suggest that the EVs are essential in regulating inflammatory signaling and immune responses in individuals coinfected with HIV and HPV, while smoking may additionally affect these interactions. Our results show that HIV infection notably boosts EV secretion and modifies cytokine composition, with greater incorporation of proinflammatory factors like IL-8, RANTES, and MCP-1, while decreasing their levels in plasma. This indicates that EVs function as storage for inflammatory cytokines, possibly promoting localized immune activation and restricting systemic inflammation.

### Limitations of the study

The major limitations of this study include a relatively small sample size and the absence of smoker-only and HPV-only control groups. The absence of smoker-only and HPV-only groups restrict interpretation of HPV-specific and smoking-specific effects. For example, the study doesn’t assert that smoking amplifies HPV effects; instead, it concludes that smoking modulates EV properties and cargo within HIV and HIV+HPV infected groups. Our study cannot distinguish biological interactions from behavioral co-occurrence. The observed smoking-HPV association may reflect shared behavioral factors, but not as proof that smoking biologically amplifies HPV effects. Additionally, all subjects had well-controlled HIV through antiretroviral treatment, which may have masked the potential effects of HPV coinfection and tobacco smoking. As a pilot study, the primary goal was to explore whether HPV and smoking further influence EV biogenesis and EV-associated inflammatory responses in individuals with HIV. Based on the findings presented above, a larger follow-up study is currently underway, which will include smoker-only and HPV-only control groups. The future study will also incorporate a longitudinal component, potentially recruiting newly HIV-infected individuals who have not yet initiated antiretroviral therapy, particularly among HPV-infected and smoker populations.

## Data Availability

The raw data supporting the conclusions of this article will be made available by the authors, without undue reservation.
